# Loss of the translational repressor 4E-BP1 promotes skin carcinogenesis

**DOI:** 10.3389/fphar.2026.1742429

**Published:** 2026-01-16

**Authors:** Yibin Zhou, Hanyu Dou, Yifeng Liu, Juan Wang, Xiaolei Ding, Taomin Huang

**Affiliations:** 1 Department of Pharmacy, Eye and ENT Hospital, Fudan University, Shanghai, China; 2 Shanghai Engineering Research Center of Organ Repair, School of Medicine, Shanghai University, Shanghai, China

**Keywords:** 4E-BP1, angiogenesis, mTORC1, proliferation, SCC

## Abstract

**Introduction:**

Skin squamous cell carcinoma (SCC) arises from dysregulated epidermal homeostasis characterized by aberrant keratinocyte proliferation and pathological angiogenesis. The eukaryotic translation initiation factor 4E‐binding protein 1 (4E‐BP1) functions as a critical suppressor of cap-dependent translation by interacting with eIF4E, thereby constraining protein synthesis and cell growth. However, its role in SCC remains elusive.

**Methods:**

To address the role of 4E‐BP1 function in SCC pathogenesis, we employed a two‐stage chemical carcinogenesis model in 4E‐BP1‐deficient mice.

**Results:**

4E‐BP1‐deficient mice exhibited a significantly increased papilloma burden compared to wild-type controls, accompanied by enhanced keratinocyte proliferation and augmented tumor vascularization. Analysis of human SCC specimens revealed elevated 4E‐BP1 phosphorylation together with increased proliferative and angiogenic markers and activation of the mTOR signaling pathway, mirroring molecular features observed in 4E‐BP1‐deficient tumors.

**Discussion:**

Collectively, these findings establish 4E‐BP1 as a tumor suppressor in skin carcinogenesis that constrains both proliferative and angiogenic processes, underscoring the contribution of dysregulated translation to SCC development.

## 1 Introduction

Non-melanoma skin cancers (NMSCs) constitute the most prevalent human malignancies worldwide, with squamous cell carcinoma (SCC) representing the second most common subtype yet exhibiting disproportionately aggressive behavior compared to basal cell carcinoma (Roky et al., 2025; Wang et al., 2023). While most SCCs are managed through surgical excision, a clinically significant subset demonstrates high-risk features, with metastatic rates approaching 5% and mortality rates of 2% (Kauvar et al., 2015). This aggressive subset faces severely limited therapeutic options, highlighting the urgent need to define molecular mechanisms that drive malignant progression (Winge et al., 2023).

SCC arises from epidermal keratinocytes through a multistep carcinogenic process in which the equilibrium between proliferation, differentiation, and tissue integrity becomes progressively disrupted (Winge et al., 2023; Li et al., 2018; Ding et al., 2015). Malignant transformation is driven by the interplay of genetic alterations and tumor-promoting environmental insults, particularly chronic ultraviolet irradiation and exposure to chemical carcinogens (Jia et al., 2024). Early events in carcinogenesis frequently involve mutations in TP53 together with alterations in CDKN2A, RAS, and NOTCH1 (Chang and Shain, 2021). At advanced stages, SCC displays an increased propensity for local invasion and distant metastasis compared with other nonmelanoma skin cancers. In addition to harboring one of the highest mutational burdens among human malignancies (Hwang et al., 2024), SCC is characterized by aberrant activation of kinase signaling networks, most notably the mechanistic target of rapamycin (mTOR) pathway which plays a pivotal role in driving malignant growth and tumor progression (Zheng et al., 2024; Mercurio et al., 2021; Amornphimoltham et al., 2005).

mTOR functions as a central regulator hub that integrates diverse extracellular cellular inputs and intracellular cues, including growth factors, nutrient availability, and stress signals to coordinate translational control and metabolic reprogramming (Battaglioni et al., 2022; Wang et al., 2022a). Aberrant activation of mTOR signaling has been documented across a wide spectrum of human malignancies (Mossmann et al., 2018). In the context of SCC, genetic activation of mTOR signaling promotes aggressive tumor phenotypes in mice, thereby establishing this pathway as a critical determinant of SCC behavior (Hertzler-Schaefer et al., 2014; Chen et al., 2019). Importantly, clinical evidence demonstrates that rapamycin treatment reduces mTOR signaling and tumor growth and resulted in significant clinical responses in head and neck SCC (Day et al., 2019). These observations underscore the central role of mTOR signaling in driving SCC progression and highlight its potential as a therapeutic target.

A critical downstream effector of mTORC1 effector is the eukaryotic initiation factor 4E-binding protein 1 (4E-BP1) (Qin et al., 2016; Dowling et al., 2010). In its hypophosphorylated state, 4E-BP1 inhibits eIF4E, thereby blocking mRNA translation and suppressing cell proliferation. By contrast, mTORC1-mediated phosphorylation relieves this inhibition, enabling synthesis of proteins required for cell proliferation, survival, and angiogenesis (Battaglioni et al., 2022; Dowling et al., 2010). Clinical evidence from human tumors supports 4E-BP1 controls tumor progression at various stages. Elevated phospho-4E-BP1 correlates with reduced survival in melanoma patients (O'Reilly et al., 2009). More than 35% of patients with head and neck SCC exhibit EIF4EBP1 gene, encoding 4E-BP1 copy loss, which is associated with altered protein expression and poor disease-free and overall survival (Wang et al., 2019; Musa et al., 2016). Thus, both hyperphosphorylation and reduced expression of 4E-BP1 converge on the same consequence, the loss of translational control and unchecked protein synthesis. Moreover, 4E-BP1 mRNA, 4EBP1 protein, and p-4E-BP1 are correlated with the activation of cancer-associated fibroblast and may serve as potential prognostic biomarkers and therapeutic targets (Du et al., 2022). Despite these insights, the direct role of 4E-BP1 in cutaneous SCC development remains unexplored.

Here, we investigated the contribution of 4E-BP1 in SCC development using 4E-BP1-deficient mice, which mimic constitutive activation of mTORC1-4E-BP1 signaling axis. We employed the classical two-stage chemical carcinogenesis model, involving 7,12-dimethylbenz(a)anthracene (DMBA) initiation followed by 12-O-tetradecanoylphorbol-13-acetate (TPA) promotion, which faithfully recapitulates the initiation and promotion phases characteristic of human SCC pathogenesis (Li and Brakebusch, 2021; Cipolat et al., 2014). We demonstrate that 4E-BP1 loss significantly increased papilloma multiplicity, accompanied by elevated keratinocyte proliferation and tumor angiogenesis. Human SCC specimens revealed mTOR pathway activation with enhanced proliferative and angiogenic markers, mirroring features in 4E-BP1-deficient tumors. These findings identify 4E-BP1 as a tumor suppressor in skin carcinogenesis, suggesting dysregulated translational control as a driver of SCC development.

## 2 Materials and methods

### 2.1 Animal models

This study utilized immunocompetent wild type (WT) and *Eif4ebp1* ^*−/−*^ mice, both on a C57BL/6 genetic background. The *Eif4ebp1* ^*−/−*^ mice were generated via CRISPR-Cas9 technology targeting the Eif4ebp1 gene (Shanghai Model Organisms Center, China). Wild-type littermates were used as controls to minimize genetic variability. All mice were housed in a pathogen-free animal facility under controlled environmental conditions, including a 12-h light/dark cycle, a constant temperature of 22 °C ± 1 °C, and humidity of 50%–60%. They were fed a standard chow diet with *ad libitum* access to water and food. Genotyping was conducted through polymerase chain reaction (PCR) analysis of genomic DNA extracted from tail tips. The sequences of primers used were as follows: P1, 5′-TTC TGC CAC CGT CAT CCC TA-3′; P2, 5′-AGC TAC CGA ACC CCT CGA AT-3′; P3, 5′-AAT CGG AGA GTT CTG CCA CC-3′; P4, 5′-GGA TCC CGA CGT ATC CTC CA-3′. All animal experiments were approved by the Institutional Animal Care and Use Committee of Shanghai University (ECSHU 2022-100).

### 2.2 Carcinogenesis model

To investigate the role of 4E-BP1 in skin carcinogenesis, we employed a two-stage chemical carcinogenesis model. The dorsal hair of the mice was removed using an electric shaver, and shaving was performed weekly to maintain a hair-free area for chemical application and tumor observation. In the initiation phase, a single dose of 200 µL of 0.25 mM DMBA (Cat. No. 1022, Selleck) dissolved in acetone was applied to the dorsal skin of each mouse to induce the genetic mutations required for tumor initiation. One week later, the promotion phase was initiated by topical application of TPA (Cat. No. P8139, Sigma-Aldrich; 6.25 µg in 200 µL acetone per application) to the dorsal skin twice weekly for a total of 21 weeks to stimulate tumor development. Tumor incidence, number, and size were recorded weekly throughout the promotion phase, and at the end of treatment in week 21, skin lesions were excised, fixed, and processed for hematoxylin and eosin (H&E) staining to evaluate tumor morphology and progression.

### 2.3 TPA acute stimulation model

To evaluate the acute effects of TPA on skin tissues and assess early molecular and histological responses, a short-term stimulation protocol was performed in both control and *Eif4ebp1* ^*−/−*^ mice. A single topical application of 6.25 µg of TPA dissolved in 200 µL of acetone was applied to the dorsal skin of each mouse, while mice treated with 200 µL of acetone alone served as negative controls. Forty-eight hours after treatment, skin tissues were harvested and processed for H&E staining to evaluate acute epidermal hyperplasia and inflammatory responses. The thickness of the epidermis and dermis was measured on histological sections to quantify the extent of hyperplasia and inflammation, thereby enabling comparisons between 4E-BP1-deficient and wild-type mice in response to TPA stimulation.

### 2.4 Human tissues collection and preparation

Human SCC tissues were obtained from Eye and ENT Hospital of Fudan University, Shanghai, China, following ethical approval from the Institutional Review Board (IRB) (2024233). Formalin-fixed, paraffin-embedded tumor samples were collected from five patients diagnosed with SCC. Tumor tissues were sectioned into 4-µm-thick slices using a rotary microtome and mounted on positively charged glass slides for histological analyses.

### 2.5 Western blot

Cells and tissues were lysed using radioimmunoprecipitation assay (RIPA) buffer (Cat. No. P0013K, Beyotime) supplemented with protease inhibitors (Cat. No. ST506, Beyotime) and phosphatase inhibitors (Cat. No. G2007, Servicebio). Protein concentrations were measured using a BCA assay (Cat. No. P0010, Beyotime). Equal amounts of protein (20 μg) were separated by SDS-PAGE on Tris-polyacrylamide gels and transferred onto PVDF membranes. Following blocking with 5% non-fat milk in TBST, membranes were incubated with primary antibodies and subsequently with the appropriate secondary antibodies. The following primary antibodies were used: 4E-BP1 (Cat. No. 9644, Cell Signaling Technology), GAPDH (Cat. No. 10494-1-AP, Proteintech).

### 2.6 Histological analysis

H&E staining was performed on paraffin-embedded sections of human mouse skin and SCC tissues to evaluate histopathological features, including epidermal hyperplasia, dermal infiltration, and overall tumor morphology. Paraffin-embedded sections were deparaffinized in xylene and rehydrated through graded ethanol solutions. The slides were then stained with hematoxylin for 5 min, rinsed in running tap water, and counterstained with eosin for 1 min. After dehydration through an ascending series of ethanol and clearing in xylene, slides were mounted with a synthetic resin medium. Stained sections were examined under a light microscope, and images were acquired for morphological assessment to identify key histological features in both mouse skin and human SCC tissues. For Masson trichrome staining, deparaffinized sections were stained with Weigert’s iron hematoxylin, followed by Biebrich scarlet–acid fuchsin and aniline blue to visualize collagen fibers.

### 2.7 Immunohistochemistry (IHC)

For IHC analyses, paraffin-embedded skin sections were deparaffinized with xylene and rehydrated through graded ethanol solutions. Antigen retrieval was performed by heating the sections in citrate buffer (pH 6.0) for 20 min. Endogenous peroxidase activity was quenched with 3% hydrogen peroxide, and sections were blocked with 5% normal serum for 30 min to minimize non-specific binding. The sections were then incubated overnight at 4 °C with primary antibodies targeting p-4E-BP1 Thr 37/46 (Cat. No. 2855, Cell Signaling Technology), Ki67 (Cat. No. ab15580, Abcam), and VEGFA (Cat. No. ab51745, Abcam). After washing with PBS, sections were incubated with horseradish peroxidase (HRP)-conjugated secondary antibodies for 1 h at room temperature. Signal development was performed using a diaminobenzidine (DAB) substrate, followed by counterstaining with hematoxylin. The stained slides were dehydrated, mounted with a cover slip, and examined under a light microscope. Quantification of positively stained cells and staining intensity was conducted using ImageJ software to assess the expression levels of the targeted proteins. Ki67-positive cells were quantified in the epidermal basal layer or tumor nests, whereas VEGF-A expression was evaluated in the epidermis of normal skin and the tumor epithelium of papillomas and SCC lesions.

### 2.8 Immunofluorescence (IF)

For IF analyses, fresh skin tissues were embedded in optimal cutting temperature (OCT) compound, frozen in liquid nitrogen, and stored at −80 °C. Frozen sections were cut at a thickness of 8 µm using a cryostat and air-dried at room temperature for 10 min. The sections were fixed with 4% paraformaldehyde for 15 min, followed by three washes with PBS. Non-specific binding was blocked with 5% bovine serum albumin (BSA) in PBS for 1 h at room temperature. Sections were then incubated overnight at 4 °C with primary antibodies targeting p-4E-BP1 Thr 37/46 (Cat. No. 2855, Cell Signaling Technology), p-S6 Ser240/244 (Cat. No. 5364, Cell Signaling Technology), CD31 (Cat. No. 557355, Becton, Dickinson and Company) and Ki67 (Cat. No. ab15580, Abcam), diluted in blocking buffer. After washing, sections were incubated with fluorescently labeled secondary antibodies for 1 h in the dark. Nuclei were counterstained with DAPI, and sections were mounted using an anti-fade reagent. Fluorescent signals were visualized and imaged using a confocal laser scanning microscope, and quantitative analysis, including mean fluorescence intensity and protein colocalization, was performed using ImageJ software. Ki67^+^ cells were counted in the basal cells of epidermis, and CD31^+^ areas were quantified in the dermis and tumor stroma area.

### 2.9 Statistical analysis

All statistical analyses were performed using GraphPad Prism 9 (GraphPad Software Inc.). Quantitative data are presented as mean ± standard error of the mean (SEM) or mean ± standard deviation (SD), unless otherwise specified. Statistical significance was set at p < 0.05. Comparisons between two groups were conducted using the two-tailed unpaired Student’s t-test for normally distributed data. For comparisons among three or more groups, one-way ANOVA followed by Tukey’s *post hoc* test was applied for normally distributed data. Two-way ANOVA was used when analyzing multiple factors, and repeated measures ANOVA with Bonferroni correction was applied for time-course data. Tumor incidence was analyzed using the Log-rank (Mantel-Cox) test. For histological quantifications, staining intensities from IHC and IF were measured using ImageJ software, with group differences analyzed by one-way ANOVA as appropriate. All results were derived from a minimum of three independent experiments.

## 3 Results

### 3.1 Generation of 4E-BP1 deficient mice

**FIGURE 1:**
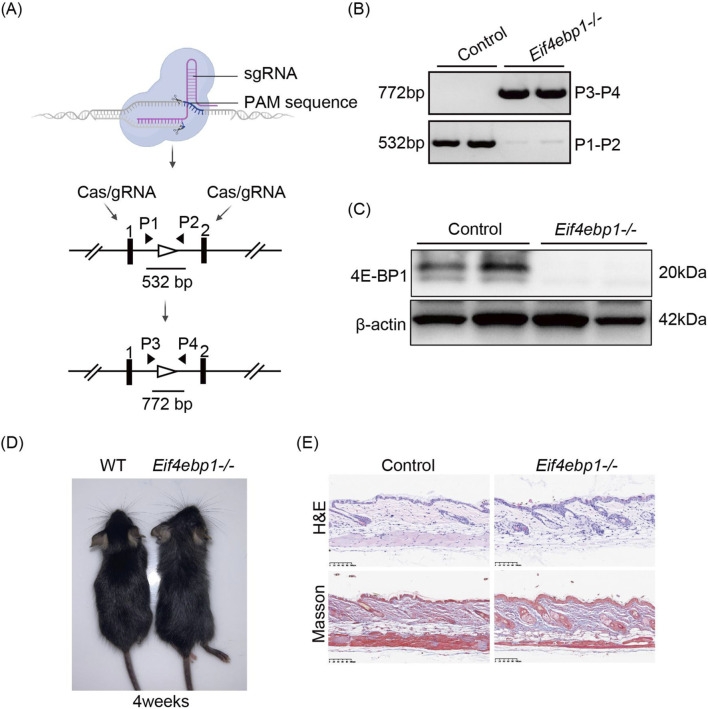
Generation of *Eif4ebp1* ^*−/−*^ mouse model. **(A)** CRISPR/Cas9-based strategy for generating *Eif4ebp1* ^*−/−*^ mice. **(B)** PCR analysis of *Eif4ebp1* ^*−/−*^ mice genotyping. **(C)** Western blot analysis of 4E-BP1 protein expression in control and *Eif4ebp1* ^*−/−*^ mice. **(D)** Representative images of WT and *Eif4ebp1* ^*−/−*^ mice. **(E)** Representative H&E-stained sections (top) and Masson trichrome staining (bottom) illustrating collagen deposition in skin tissues from control and *Eif4ebp1* ^*−/−*^ mice under baseline conditions. Scale bar, 100 μm.

Mice carrying a targeted deletion of *Eif4ebp1* on C57BL/6 background were generated using CRISPR/Cas9-mediated genome editing (Figure 1A). To validate the efficiency of 4E-BP1 deletion, the gene recombination and expression were validated at the genetic, molecular, and phenotypic levels. PCR genotyping with allele-specific primers confirmed successful deletion of *Eif4ebp1* (Figure 1B). Consistently, Western blot analysis of dorsal skin extracts demonstrated complete loss of 4E-BP1 protein in *Eif4ebp1* ^*−/−*^ mice compared with controls (Figure 1C), confirming effective disruption of 4E-BP1 expression.

Consistent with previous observation, *Eif4ebp1* ^*−/−*^ mice displayed no overt developmental or health abnormalities, and gross skin morphology remained intact (Figure 1D) (Le Bacquer et al., 2019; Tsukiyama-Kohara et al., 2001). Histological analysis of skin sections stained with H&E revealed comparable epidermal thickness or dermal architecture between genotypes under homeostatic conditions (Figure 1E). Together, these results demonstrate efficient depletion of 4E-BP1 and establish *Eif4ebp1* ^*−/−*^ mice as a suitable model to investigate its function in skin homeostasis and carcinogenesis.

### 3.2 Loss of 4E-BP1 promotes skin tumorigenesis

**FIGURE 2:**
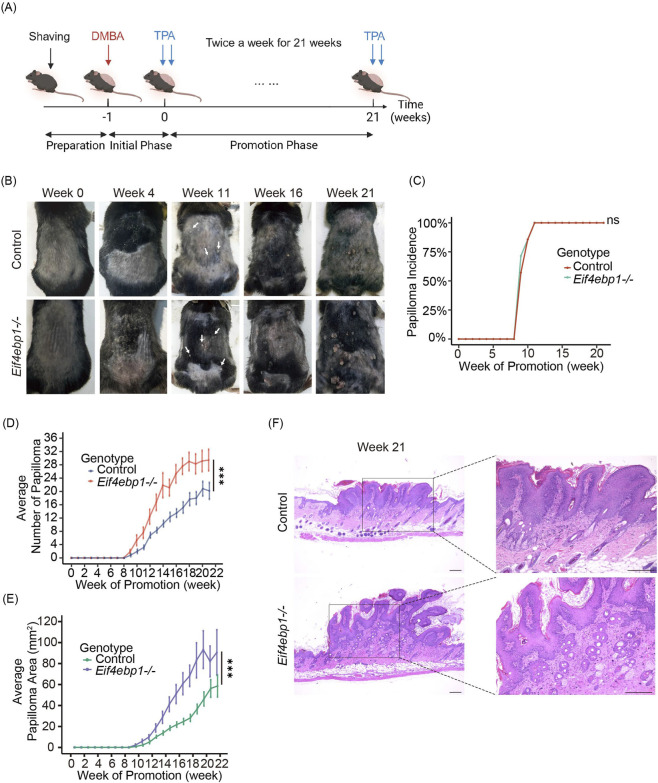
4E-BP1 loss enhances skin tumor development. **(A)** Timeline of chemical-induced skin tumor formation in mice. **(B)** Representative macroscopic image in the skin at indicated weeks following DMBA/TPA administration. **(C)** Tumor incidence over time for control and *Eif4ebp1* ^*−/−*^ mice (n = 7). **(D)** Average tumor multiplicity (number of tumors per mouse) over time (n = 7). **(E)** Average tumor size over time (n = 7). **(F)** Representative H&E images of the tumor at 21 weeks after DMBA/TPA administration. Scale bar, 250 μm. ns, not significant (Log-rank test for incidence in **(C)**); *p < 0.05, **p < 0.01, ***p < 0.001 by two-way repeated-measures ANOVA (for tumor multiplicity and size in (D, E)).

To explore whether 4E-BP1 regulates susceptibility to skin carcinogenesis, we subjected *Eif4ebp1* ^*−/−*^ and wild-type control mice to the classical two-stage carcinogenesis protocol combining DMBA initiation with repeated TPA promotion (Figure 2A). Papillomas appeared with similar latency in both genotypes, first becoming visible after approximately 8 weeks of TPA promotion (Figures 2B,C), consistent with previous reports (Li and Brakebusch, 2021). This finding indicates that loss of 4E-BP1 does not influence tumor initiation.

In contrast, striking differences emerged during tumor progression. *Eif4ebp1* ^*−/−*^ mice developed significantly more papillomas and exhibited a greater cumulative tumor burden compared with controls (Figures 2B,D,E). These differences became evident after week 13 and persisted throughout the experimental period. By week 21, tumor multiplicity and total tumor load in *Eif4ebp1* ^*−/−*^ mice were markedly elevated compared with wild-type animals.

Histopathological analysis further revealed enhanced tumor aggressiveness in *Eif4ebp1* ^*−/−*^ mice. Lesions displayed expansive papillomatous growth with pronounced epidermal hyperplasia and dermal invasion (Figure 2F). These tumors exhibited increased inflammatory infiltrates and disrupted tissue architecture, features consistent with enhanced malignant potential. In contrast, papillomas from wild-type mice were generally smaller, more circumscribed, and maintained more regular histological organization. Collectively, these findings demonstrate that while 4E-BP1 loss does not alter tumor initiation, it markedly accelerates papilloma development and progression, establishing 4E-BP1 as a suppressor of chemically induced skin tumorigenesis.

### 3.3 4E-BP1 loss increases tumor cell proliferation and angiogenesis

**FIGURE 3:**
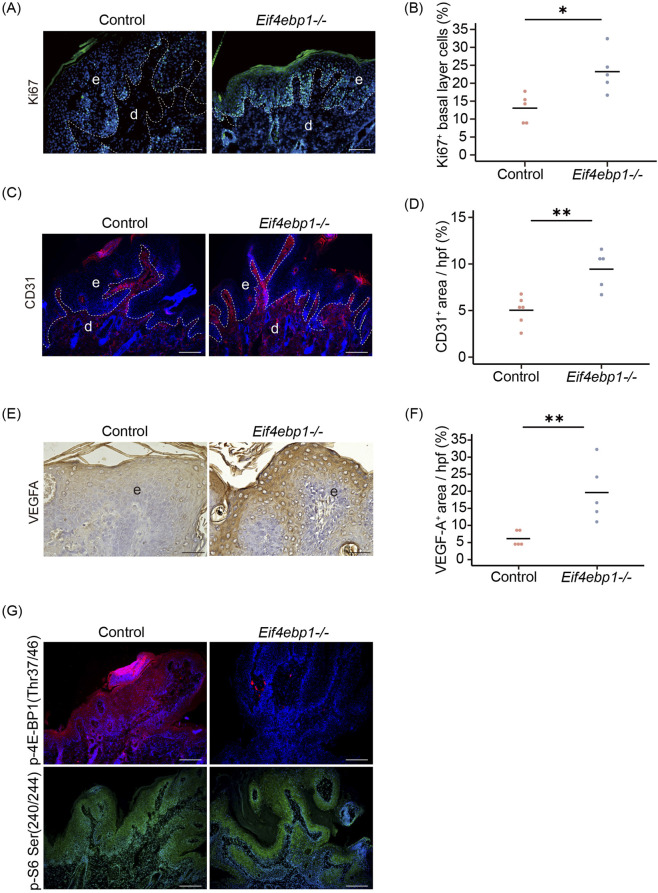
Loss of 4E-BP1 promotes cell proliferation and angiogenesis during tumorigenesis. **(A)** Representative IF images of Ki67 staining in control and *Eif4ebp1* ^*−/−*^ tumor tissues. Scale bar, 200 μm. **(B)** Quantification of the percentage of Ki67^+^ cells in control and *Eif4ebp1* ^*−/−*^ tumors (n = 5). **(C)** Representative IF images of CD31 staining in control and *Eif4ebp1* ^*−/−*^ tumor tissues. Scale bar, 200 μm. **(D)** Quantification of CD31^+^ area per hpf (n = 5–6). **(E)** Representative IHC images of VEGF-A staining in control and *Eif4ebp1* ^*−/−*^ tumor tissues. Scale bar, 50 μm. **(F)** Quantification of VEGF-A^+^ area per hpf (n = 5). **(G)** Representative IF images of p-4E-BP1 Thr37/46 (undetectable in Eif4ebp1^*−/−*^ tumors) and p-S6 Ser240/244 staining in control and Eif4ebp1^*−/−*^ tumor tissues. Scale bar, 200 μm. Ki67 (green) with DAPI nuclear counterstaining (blue); CD31 (red) with DAPI (blue); and phosphorylated 4E-BP1 (red) with DAPI (blue). The dashed line indicates the junction between the epidermis and dermis. d, dermis; e, epidermis; hpf, per high- power field. *p < 0.05, **p < 0.01, ***p < 0.001 by Student’s unpaired two-tailed t-test.

To gain mechanistic insights into the accelerated tumor progression observed in *Eif4ebp1* ^*−/−*^ mice, we performed IHC and IF analyses of skin tumor tissues collected after the 21-week carcinogenesis protocol, focusing on cellular proliferation, angiogenesis, and mTOR downstream signaling activity. Ki67 staining revealed a significantly elevated proliferative index in *Eif4ebp1* ^*−/−*^ tumors compared with wild-type controls (Figures 3A,B). The density of Ki67^+^ keratinocytes within the tumor epithelium was substantially higher in *Eif4ebp1* ^*−/−*^ lesions, indicating enhanced mitotic activity and a more aggressive growth phenotype.

To further evaluate angiogenesis, we next analyzed CD31 and VEGF-A expression. CD31 staining demonstrated markedly increased micro-vessel density in *Eif4ebp1* ^*−/−*^ tumors (Figures 3C,D), indicating enhanced angiogenic responses. Consistently, VEGF-A staining revealed stronger expression in *Eif4ebp1* ^*−/−*^ tumors compared with controls (Figures 3E,F), suggesting upregulation of pro-angiogenic signaling and further supporting the notion that 4E-BP1 deficiency promotes neovascularization in the tumor microenvironment.

We then assessed whether these phenotypic alterations were associated with changes in mTOR pathway activity. IF analysis revealed strong cytoplasmic staining for phosphorylated 4E-BP1 (p-4E-BP1) in wild-type tumors, whereas, as expected, no signal was detected in *Eif4ebp1* ^*−/−*^ lesions (Figure 3G). Notably, phosphorylated S6 ribosomal protein (p-S6), another canonical mTORC1 effector, remained robustly expressed in both genotypes (Figure 3G), indicating that other mTORC1 downstream branches remain active in the absence of 4E-BP1. Collectively, these data demonstrate that 4E-BP1 acts as a key brake on cell proliferation and angiogenesis during skin carcinogenesis. Loss of 4E-BP1 relieves translational control, fostering hyperproliferative epithelial growth and an angiogenic microenvironment that together drive accelerated tumor progression.

### 3.4 4E-BP1 deficiency amplifies early responses to TPA stimulation

**FIGURE 4:**
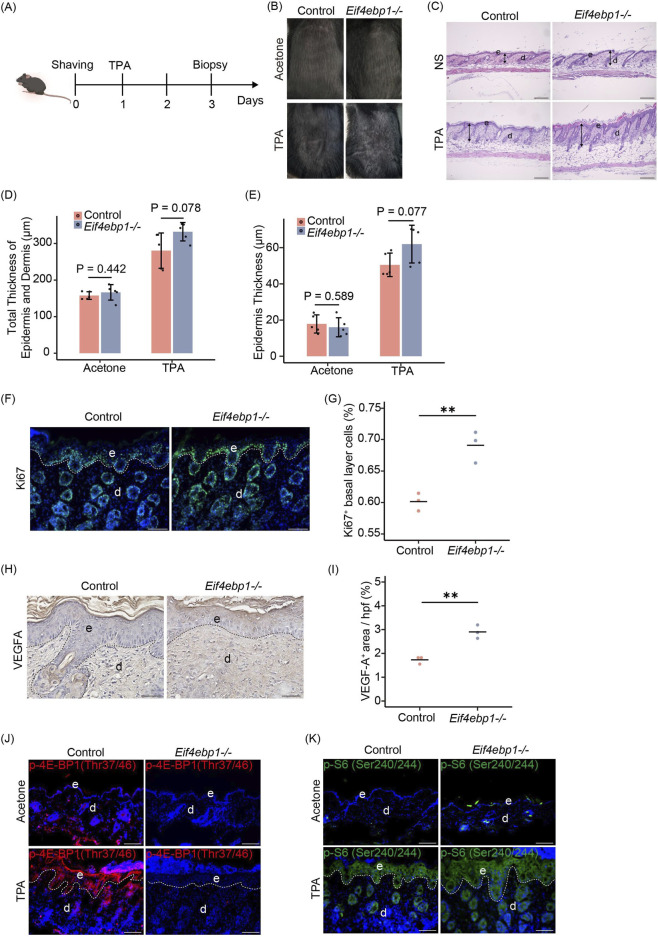
4E-BP1 loss enhances early TPA-induced responses. **(A)** Experimental timeline of short-term (48 h) TPA stimulation in mouse skin. **(B)** Dorsal skin appearance following acute TPA stimulation. **(C–E)** H&E staining showing epidermal hyperplasia and dermal inflammation, with quantitative measurements of thickness (n = 5). Scale bar, 200 μm. **(F)** Representative IF images of Ki67 staining showing keratinocyte proliferation. Scale bar, 100 μm. **(G)** Quantitative analysis of fluorescence intensity (n = 3). **(H)** Representative IHC images of VEGF-A staining indicating angiogenic activity. Scale bar, 50 μm. **(I)** Quantitative analysis of VEGF-A expression levels (n = 3). **(J)** IF staining of p-4E-BP1 Thr37/46 in skin sections from mice treated with TPA for 48 h. Scale bar, 100 μm. **(K)** IF staining of p-S6 Ser240/244 in skin sections from mice treated with TPA for 48 h. Scale bar, 100 μm. d, dermis; e, epidermis. *p < 0.05, **p < 0.01, ***p < 0.001 by Student’s unpaired two-tailed t-test.

To examine the early molecular and histological responses to tumor promotion, we performed a short-term (48 h) TPA stimulation experiment in wild-type and *Eif4ebp1* ^*−/−*^ mice (Figure 4A). We observed that the dorsal skin of mice developed pronounced erythema, induration, and thickening 48 h after TPA treatment (Figure 4B).

Histological analysis of H&E-stained sections revealed epidermal hyperplasia and dermal inflammation in both groups. These alterations were more pronounced in *Eif4ebp1* ^*−/−*^ mice, which displayed a trend toward increased epidermal thickness compared to controls (Figures 4C–E). IF staining for Ki67 showed increased keratinocyte proliferation in both genotypes, with *Eif4ebp1* ^*−/−*^ mice displaying significantly more Ki67-positive nuclei in the epidermis (Figures 4F,G). IHC analysis of VEGF-A revealed enhanced angiogenesis in response to TPA in both groups, while *Eif4ebp1* ^*−/−*^ mice exhibited markedly stronger VEGFA expression in the epidermis and dermis, especially in areas with dense inflammatory infiltration (Figures 4H,I). Similarly, IF analysis showed a pronounced accumulation of p-4E-BP1 and p-S6 in the epidermis following TPA stimulation (Figures 4J,K), indicating robust activation of the mTORC1 signaling pathway. Together, these findings indicate that loss of 4E-BP1 amplifies the early proliferative, angiogenic responses of the skin to acute TPA stimulation.

### 3.5 Phosphorylated 4E-BP1 is increased human SCC tissues

**FIGURE 5:**
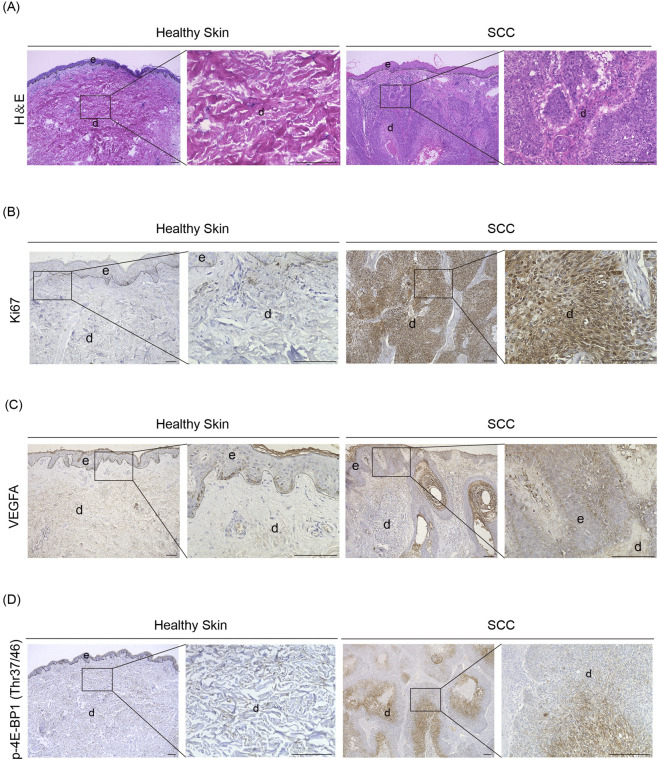
Enhanced 4E-BP1 phosphorylation in human SCC tissues. **(A)** Representative H&E staining images of human healthy skin and SCC tissues. Scale bar, 200 μm. **(B)** Representative IHC images of Ki67 staining in human healthy skin and SCC tissues. Scale bar, 100 μm. **(C)** Representative IHC images of CD31 staining in human healthy skin and SCC tissues. Scale bar, 100 μm. **(D)** Representative IHC images of p-4E-BP1 Thr37/46 staining in human healthy skin and SCC tissues. Scale bar, 200 μm. d, dermis; e, epidermis.

To determine whether our findings in *Eif4ebp1* ^*−/−*^ mice, which mimic constitutive activation of eIF4E-dependent translation, are relevant to human disease, we examined phosphorylated 4E-BP1 levels in human SCC specimens. H&E staining revealed characteristic features of SCC, including irregular nests of atypical keratinocytes invading the dermis, keratin pearl formation, prominent keratinization, and marked nuclear atypia with frequent mitotic figures. The surrounding stroma exhibited dense inflammatory infiltration (Figure 5A). Ki67 staining demonstrated high proliferative activity, particularly in keratinocytes at the invasive front of tumor nests (Figure 5B). VEGF-A expression was markedly elevated in peritumoral stromal regions, indicating active angiogenesis within the tumor microenvironment (Figure 5C). Importantly, phosphorylated 4E-BP1 displayed strong cytoplasmic expression in tumor epithelial cells across all patient samples compared with healthy skin tissues, consistent with robust activation of the mTORC1-4E-BP1 signaling axis (Figure 5D).

## 4 Discussion

In this study, we identify the translational regulator 4E-BP1 as a critical modulator of SCC growth. Using a genetic mouse model, we show that loss of 4E-BP1 accelerates tumor progression, characterized by increased keratinocyte proliferation, enhanced angiogenesis, and heightened mTOR pathway activation. These features were mirrored in human SCC, where phosphorylated 4E-BP1 was consistently elevated in tumor epithelial cells. The concordance between mouse and human disease indicates the robustness of our findings and underscores the translational value of targeting mTOR–4E-BP1 signaling in SCC. By linking protein synthesis control to the malignant behavior of keratinocytes, our work provides important mechanistic insights into squamous carcinogenesis.

Cap-dependent translation is increasingly recognized as a central mechanism in the induction and maintenance of the transformed phenotype (Mamane et al., 2004; Thoreen et al., 2012). 4E-BP1 serves as a key regulator of this process by inhibiting translation initiation through its interaction with the cap-binding protein eIF4E (Thoreen et al., 2012). The mTORC1-4E-BP1 axis has emerged as a critical effector of oncogenic signaling pathways that are commonly deregulated in human cancers (Guertin and Sabatini, 2007; Madhu and Kannan, 2025; Wang et al., 2022b). In human SCC, which almost invariably harbors mutations in oncogenes and tumor suppressors, many of these alterations converge on translational control (Dotto and Rustgi, 2016). Previous studies have shown that 4E-BP1 mediates the oncogenic effects of such mutations by regulating the translational output of key effector genes (She et al., 2010). Notably, previous studies have implicated 4E-BP1 in other squamous malignancies, particularly head and neck squamous cell carcinoma (HNSCC). Wang et al. demonstrated that 4E-BP1 functions as a tumor suppressor in HNSCC, where its expression is often reduced or functionally inactivated by hyperphosphorylation (Wang et al., 2019; Musa et al., 2016). While these studies provided crucial insights, the specific functional role of 4E-BP1 in cutaneous SCC development has remained less defined, and direct *in vivo* genetic evidence in an immunocompetent skin model has been lacking. In this context, our study provides direct genetic evidence that loss of 4E-BP1 facilitates tumor progression. *Eif4ebp1* ^*−/−*^ mice developed significantly larger and more advanced tumors compared with wild-type controls, despite similar tumor incidence, indicating that 4E-BP1 restrains tumor growth primarily at the level of progression rather than initiation. Histological analyses further revealed that tumors lacking 4E-BP1 were characterized by pronounced epidermal hyperplasia and augmented angiogenesis, thereby establishing a link between translational control, epithelial proliferation, and the tumor-associated microenvironment.

Angiogenesis is a hallmark of tumor progression, and translational control has emerged as a critical regulator of this process (Lugano et al., 2020; Welti et al., 2013). VEGF-A, a major angiogenic factor, is highly dependent on cap-dependent translation and thus particularly sensitive to regulation by the eIF4E–4E-BP1 axis (Dodd et al., 2015). In our study, *Eif4ebp1* ^*−/−*^ tumors displayed markedly increased VEGF-A expression and pronounced angiogenic responses, as evidenced by histological and IHC analyses. These findings align with the concept that loss of 4E-BP1 relieves translational repression of VEGF-A, thereby enhancing neovascularization within the tumor microenvironment. By facilitating the sustained delivery of oxygen and nutrients, this angiogenic switch may underline the observed increase in tumor size and progression in the absence of 4E-BP1. Together, these results highlight VEGF-A as a functionally relevant translational target of eIF4E and position 4E-BP1 as a key node linking oncogenic signaling pathways to the vascular remodeling that supports tumor growth.

Beyond its canonical role in regulating cap-dependent translation, emerging evidence suggests that 4E-BP1 exerts broader cellular functions (Qin et al., 2016; Morita et al., 2013). Recent studies have implicated 4E-BP1 in the regulation of mitochondrial activity and metabolic adaptation, processes that are increasingly recognized as integral to tumor cell survival and progression (He et al., 2023). Although our study primarily links 4E-BP1 loss to translational de-repression of growth- and angiogenesis-related transcripts such as VEGF-A, it remains conceivable that mitochondrial dysfunction may further contribute to the enhanced tumor progression observed in *Eif4ebp1* ^*−/−*^ mice. Addressing these non-translational functions will be important for a more comprehensive understanding of 4E-BP1 biology in cancer.

While ultraviolet (UV) radiation is the primary etiological factor for human cSCC, often characterized by *TP53* signature mutations, the DMBA/TPA two-stage chemical carcinogenesis model employed in this study serves as a pivotal platform for dissecting the multistep nature of epithelial malignancies. For decades, this model has been widely used to resolve stem cell dynamics during normal homeostasis and oncogenic transformation (Abel et al., 2009; Taylor et al., 2024). Its key advantage lies in its ability to faithfully recapitulate the dynamic continuum of carcinogenesis—from normal skin to benign precursor lesions (papillomas), and ultimately to malignant cSCC and metastasis—which remains challenging to model stably with UV irradiation alone. Importantly, these tumors are induced by environmental factors rather than genetic engineering alone, harboring a high burden of somatic mutations that resemble the driver mutations found in human tumors (McCreery et al., 2015). Furthermore, the tumors develop *in situ*, preserving natural mouse biology and immune interactions within an immunocompetent host, rather than relying on immunodeficient models. In this context, the *Eif4ebp1* ^*−/−*^ mouse model provides a unique perspective for exploring the regulatory role of translational control in cSCC progression. Despite the differences in initiating events between chemical (*H-ras* driven) and UV (*TP53* driven) carcinogenesis, the aberrant activation of the mTORC1 signaling axis is a convergent molecular feature in both etiologies. Thus, our findings regarding 4E-BP1 function likely have broad implications for cutaneous SCC arising from diverse environmental insults.

A limitation of our study is the use of a systemic Eif4ebp1 knockout model. This approach does not allow us to dissect tissue- or cell type–specific contributions of 4E-BP1, particularly in the tumor epithelium versus the stromal or immune compartments of the skin (Schütz et al., 2023). Given the prominent cell proliferation and angiogenic responses observed, future studies employing single-cell analysis and conditional or inducible knockout strategies will be essential to clarify the cell-autonomous versus microenvironmental roles of 4E-BP1 during skin carcinogenesis (Zou et al., 2023). Such approaches may also help determine whether targeting the eIF4E–4E-BP1 axis could provide therapeutic benefit without interfering with homeostatic functions in non-tumor tissues.

Taken together, our findings establish 4E-BP1 as a critical regulator of tumor progression in cutaneous squamous cell carcinoma by restraining both proliferative and angiogenic programs. The data provide mechanistic and translational support for considering the eIF4E–4E-BP1 axis as a therapeutic vulnerability in SCC and highlight the importance of integrating translational control into our understanding of tumor biology.

## Data Availability

The raw data supporting the conclusions of this article will be made available by the authors, without undue reservation.
